# Dust emission source characterization for visibility hazard assessment on Lordsburg Playa in Southwestern New Mexico, USA

**DOI:** 10.1186/s40677-020-00171-x

**Published:** 2020-12-10

**Authors:** R. Scott Van Pelt, John Tatarko, Thomas E. Gill, Chunping Chang, Junran Li, Iyasu G. Eibedingil, Marcos Mendez

**Affiliations:** 1 USDA Agricultural Research Service, 302 W. Interstate 20, Big Spring, TX USA; 2 USDA Agricultural Research Service, Ft. Collins, CO USA; 3 Department of Geological Sciences, and Environmental Science & Engineering Program, University of Texas at El Paso, El Paso, TX USA; 4 Hebei Normal University, Shijiazhuang, Hebei P. R. China; 5 Department of Geosciences, The University of Tulsa, Tulsa, OK USA; 6 Environmental Science and Engineering Program, University of Texas at El Paso, El Paso, TX USA; 7 Department of Geological Sciences, University of Texas at El Paso, El Paso, TX USA

**Keywords:** PM_10_, Dust storms, Visibility, Highway safety, Surface emissivity, Wind erosion

## Abstract

In drylands around the world, ephemeral lakes (playas) are common. Dry, wind-erodible playa sediments are potent local and regional sources of dust and PM_10_ (airborne particles with diameters less than 10 μm). Dust clouds often cause sudden and/or prolonged loss of visibility to travelers on downwind roadways. Lordsburg Playa, in southwestern New Mexico, USA is bisected by Interstate Highway 10. Dust storms emanating from the playa have been responsible for numerous visibility-related road closures (including 39 road closures between 2012 and 2019) causing major economic losses, in addition to well over a hundred dust-related vehicle crashes causing at least 41 lost lives in the last 53 years. In order to improve understanding of the surfaces responsible for the dust emissions, we investigated the critical wind friction velocity thresholds and the dust emissivities of surfaces representing areas typical of Lordsburg Playa’s stream deltas, shorelines, and ephemerally flooded lakebed using a Portable In-Situ Wind ERosion Laboratory (PI-SWERL). Mean threshold friction velocities for PM_10_ entrainment ranged from less than 0.30 m s^− 1^ for areas in the delta and shoreline to greater than 0.55 m s^− 1^ for ephemerally flooded areas of the lakebed. Similarly, we quantified mean PM_10_ vertical flux rates ranging from less than 500 μg m^− 2^ s^− 1^ for ephemerally flooded areas of lakebed to nearly 25,000 μg m^− 2^ s^− 1^ for disturbed delta surfaces. The unlimited PM_10_ supply of the relatively coarse sediments along the western shoreline is problematic and indicates that this may be the source area for longer-term visibility reducing dust events and should be a focus area for dust mitigation efforts.

## Introduction and background

**Fig. 1:**
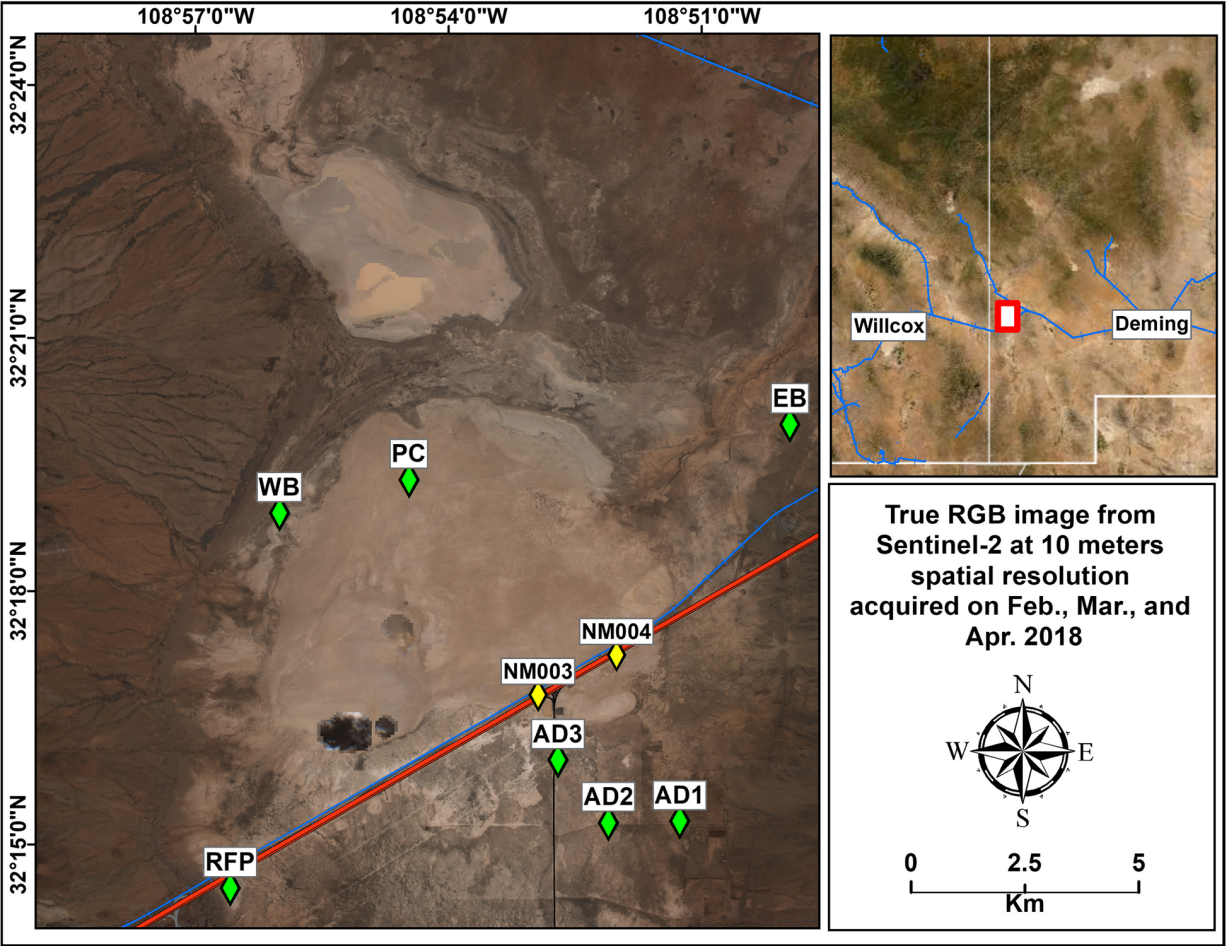
Satellite image of Lordsburg Playa dissected by Interstate 10 showing the locations (green diamonds) of surfaces that were tested with the PI-SWERL as listed in Table 1 and the NMDOT weather stations (NM003 and NM004) (yellow diamonds)

Semiarid and arid regions of the world are disproportionate sources of windblown sand (Pye and Tsoar 2009) and dust aerosols (Prospero et al. 2002; Zobeck and Van Pelt 2006). Playas (dry or intermittently-wetted lake beds in internal drainage basins), being flat, windswept, and unvegetated, are prominent source areas of dust storms globally (Prospero et al. 2002) including the Chihuahuan Desert of southwest North America (Baddock et al. 2011a, 2011b). One such playa is Lordsburg Playa in Hidalgo County, southwestern New Mexico, USA, just east of the Arizona state line. According to Botkin and Hutchinson (2020), at least 120 dust events affected the Lordsburg Playa during the eight years from 2012 to early 2020. Lordsburg Playa is not only one of the sources of the most intense dust storms in the Chihuahuan Desert (Rivera-Rivera et al. 2010), but is also crossed by Interstate Highway 10 (Fig. 1), the southernmost transcontinental highway in the American Interstate Highway System, and a major transportation artery extending from Florida to California. Approximately 15,000 vehicles per day, about 30% of which were trucks, crossed the playa on Interstate Highway 10 in 2016 (Haas 2017). The Union Pacific railroad crosses Lordsburg Playa parallel to and approximately 30 m north of the interstate.

According to the World Health Organization (2018), traffic crashes are the ninth leading cause of death worldwide. In the United States, on average, the annual death toll from motor vehicle crashes due to weather related visibility and vision hazards such as fog, smoke, dust, and blowing sand, exceeds the number of fatalities caused by other weather-related hazards including tornados, floods, tropical cyclones, and lightning (Ashley et al. 2015; Bhattachan et al. 2019). Due to the arid climate of the Chihuahuan Desert, fog, the most common weather phenomenon affecting highway visibility (Ashley et al. 2015) is rare in the Lordsburg basin.

Windblown (aeolian) dust and sand crossing transportation corridors in drylands is increasingly recognized as a direct threat to human health and safety (Goudie 2014; Baddock et al. 2013; Middleton 2017; Li et al. 2017; Zheng-chao 2018; Bhattachan et al. 2019; Davari et al. 2019). Dust and sand blowing across roadways causes sudden loss of visibility (Ashley et al. 2015) and reduced traction on the road surface (Davari et al. 2019), and together increase the likelihood for loss of vehicle control and collisions. Goudie (2014), searched newspaper clippings for 12 consecutive months in the period of 2012 to 2013, reported that dust-related fatal highway crashes happened in six states of the U.S. in a single year. Numerous case reports have been published of blowing and drifting dust and sand causing highway crashes, especially multi-vehicle incidents on high-speed roads including interstate highways (Pauley et al. 1996; Laity 2003; Goudie 2014; Deetz et al. 2016; Nicoll et al. 2020). The U.S. National Weather Service reported that dust events are the third largest weather-related cause of highway casualties in the state of Arizona, resulting in at least 157 fatalities and 1324 injuries statewide in 50 years (Lader et al. 2016).

**Fig. 2:**
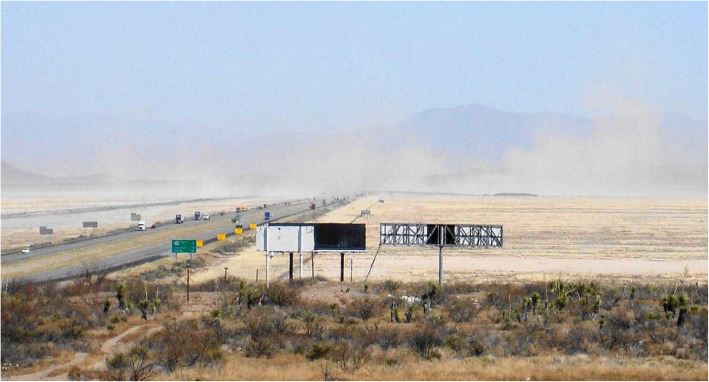
Dust clouds blowing across Interstate Highway 10 on Lordsburg Playa while the highway is open to automobile and truck traffic, March 22, 2016

Lordsburg Playa is the only dust-emitting playa crossed by an interstate highway in the southwest USA, although ~ 1000 km north of Lordsburg, Interstate Highway 80 crosses the playa of Great Salt Lake, Utah and has been the location of fatal dust-related traffic wrecks (Nicoll et al. 2020). Plumes of airborne dust and sand crossing the highway close to their source (Fig. 2) therefore represent an immediate hazard to roadway traffic due to sudden loss of visibility and resulting driver disorientation (Ashley et al. 2015; Li et al. 2017). The authors are not aware of any individual stretch of road in the southwest United States having a greater dust hazard than Interstate Highway 10 across Lordsburg Playa, where at least 117 dust/wind-related traffic crashes were recorded by public safety authorities between 1980 and 2017 (New Mexico Department of Transportation 2018). In these crashes, at least 41 dust-related traffic fatalities have occurred since 1965, including 21 deaths since 2012; seven persons were killed in one dust event in May 2014 and ten killed in 4 dust events during 2017 (Associated Press 2017; Botkin and Hutchinson 2020).

Dust and sand blowing across highways not only constitutes a direct health and safety hazard, it also causes significant economic impacts. A single traffic fatality was estimated to cost US $1.38 million in 2010 dollars when medical care, emergency services, productivity over a lifetime, insurance, workplace costs, and legal costs were considered (Blincoe et al. 2015). Beyond healthcare-related costs from crashes, aeolian dust and sand crossing highways disrupts and delays transportation and delivery of goods, services, and people, increases costs for highway maintenance and deployment of public safety personnel, and causes significant property damage; thus, it is a significant “off-site” cost of wind erosion (Pimentel et al. 1995: Baddock et al. 2013). The US Federal Highway Administration (FHWA) (2005) notes that commercial shippers and carriers value transit time at $25 to $200 per hour, depending on the value and perishability of the product being carried, so weather-related delays or detours on major transportation corridors caused by dust storms cause significant economic losses as well as health and safety hazards. Only a few studies have provided an in-depth analysis on the occurrence of such events, and little information is available to highway managers on the mitigation and management of this hazard (Li et al. 2017).

Interstate Highway 10 across Lordsburg Playa was closed to traffic for dust-related safety reasons at least 39 times between 2012 and 2019 (Botkin and Hutchinson 2020). When the highway is closed, vehicles must either wait potentially for hours until weather conditions abate, or detour 172 km and an additional > 2 h travel time onto two-lane secondary roads not designed for heavy truck traffic (ADOT 2019), causing economic losses not only via increased travel time and other logistical delays, but also by damaging pavement and bridges on the structurally-weaker alternate routes. In order to facilitate the detour’s increased traffic flow during high wind events and make this route safer, the Arizona Department of Transportation (ADOT) is planning to spend nearly US $60 million (ADOT 2019). The New Mexico Department of Transportation (NMDOT) has spent nearly US $2 million in recent years (Trent Botkin, NMDOT, personal communication) to study and mitigate the dust threat. Meanwhile other agencies including the U.S. Federal Highway Administration (FHWA), Bureau of Land Management (BLM), USDA Natural Resources Conservation Service (NRCS), and other agencies and contractors have joined with both states’ departments of transportation, working diligently together to attempt to mitigate this hazard with engineering-based and biological (vegetation-based) dust control approaches.

We initiated this study to quantify the dust emissivities of likely potential dust source surfaces surrounding and within the Lordsburg Playa. To accomplish this objective we used accepted instrumentation and methodologies to directly measure dust emissions for many representative surfaces at increasing friction velocities. With this knowledge, land managers both public and private in partnership with transportation authorities may be able to prioritize areas for control to mitigate future dust outbreaks, thus protecting human health and safety and reducing economic impacts of blowing dust and sand.

## Methods and materials

Lordsburg Playa comprises several intermittently connected ephemerally flooded dry lakebeds in the northern part of the Animas Basin within the Basin and Range physiographic province and the northwestern Chihuahuan Desert ecoregion. These dry lakebeds represent the bottom of Pleistocene Lake Animas (Allen 2005). The lakes are the termination of drainage from the Pyramid Mountains, the south and west slopes of Burro Peak, the eastern slopes of the Peloncillo Mountains, and the west and north slopes of the Animas Mountains. The largest and southernmost of the dry lake beds is sometimes named Kathrine Playa, and is the lakebed through which U.S. Interstate Highway 10 traverses. The altitude of the lakebed is approximately 1263 m above mean sea level and the climate is arid with approximately 30 cm of average annual precipitation more than half of which typically falls during the North American Monsoon between July and September. Five months of the year have average maximum temperatures in excess of 30^o^ C with June, the hottest month, having an average maximum of 35^o^ C.

Soils in the Lordsburg Playa basin are classified primarily as aridisols of the Hondale series (Fine, mixed, superactive, Thermic Natargid) in the Animas Creek delta and along the western shoreline, Playas series (Fine, mixed, superactive, Sodic Haplocambid) in the ephemerally flooded lakebed, and small areas of vertisols of the Verhalen silty clay loam series (Fine, smectitic, thermic Typic Haplotorrerts) on the eastern shoreline. Although the playa surface is saline and alkaline with occasional salt efflorescences (puffy growths of evaporite minerals on the surface) (Reynolds et al. 2007), the surface sediments are comprised of primary silicate and clay minerals with lower concentrations of evaporites (salt minerals) than many other dust-emitting playas in North America (Hibbs et al. 2000; Mitroo et al. 2019).

Vegetation of the area is typical of the lower elevation Chihuahuan Desert with dominant shrubs such as several species of saltbush (*Atriplex spp.*) and seablight (*Suaeda nigrescens* I.M. Johnst) with alkali sacaton (*Sporobolus airoides* (Torr.) Torr.) as the dominant grass in the saline areas and sand dropseed (*Sporobolus cryptandrus* (Torr.) Gray) and ring muhly (*Muhlenbergia torreyi* (Kunth) Hitchc.) in well drained sandier areas above the shoreline. Following closure of several mining districts in the Lordsburg basin during the early twentieth century, livestock grazing is the primary land use in the surrounding area.

We conducted tests on surface dust emissivity using a Portable In-Situ Wind ERosion Laboratory (PI-SWERL) (Etyemezian et al. 2007) in March 2018. The PI-SWERL is a computer-controlled aspirated cylindrical chamber approximately 30 cm in diameter that entrains dust by rotating a metallic ring a few centimeters above the soil surface, creating the shear stress necessary to entrain loose particles. The cylindrical chamber is aspirated with 1.67 l s^− 1^ of filtered air and a portion of the exhaust is continuously drawn into a DustTrak, a fast response nephelometer (Model 8530, TSI Instruments, Shoreview, Minnesota, USA) to measure PM_10_ (airborne particles with diameter smaller than 10 μm) concentrations. The PI-SWERL has been shown to provide data on dust emissions and surface erodibilities very similar to a larger linear wind tunnel (Sweeney et al. 2008) and has proven useful for estimating dust emissivities at other playas including Yellow Lake, Texas (Sweeney et al. 2016), the Salton Sea, California (King et al. 2011), and several playa surfaces in Namibia (Von Holdt et al. 2019).

**Fig. 3:**
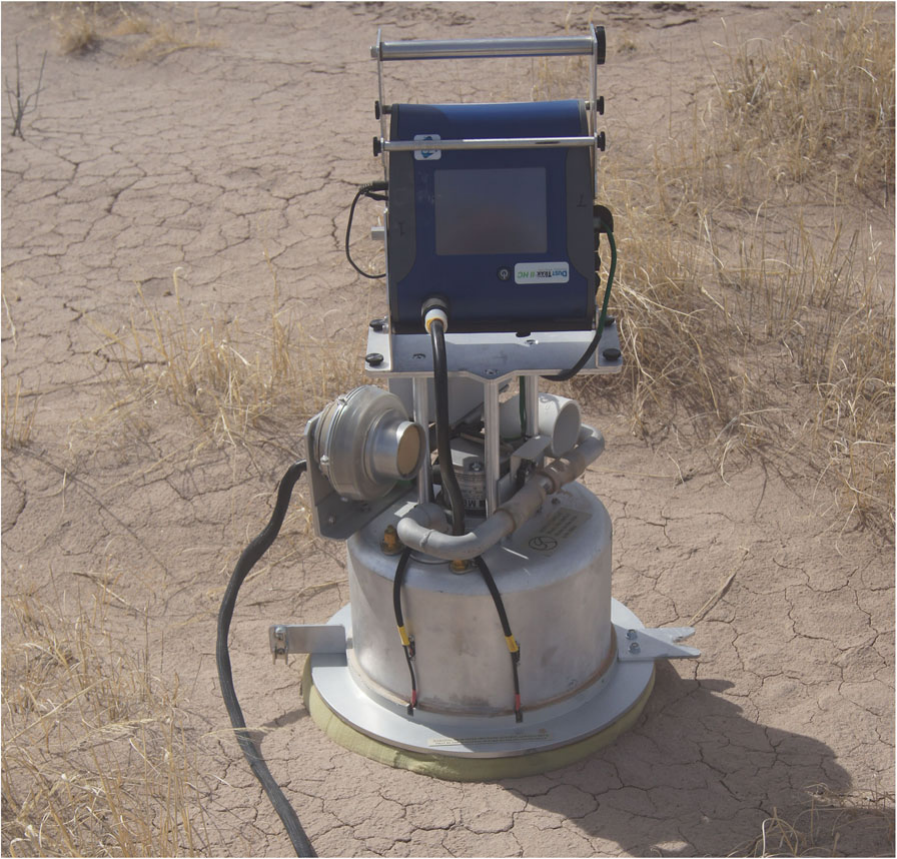
PI-SWERL in test configuration on an undisturbed surface at the eastern beach area (EPL)

For tests on the Lordsburg Playa surfaces, we set the PI-SWERL on a surface typical of the surrounding area (Fig. 3) and initiated a nine-minute (540 s) hybrid test (Table 1). The hybrid PI-SWERL test intervals and RPMs were determined in preliminary tests (unpublished) on a wide variety of soil surfaces throughout the US to provide consistency for comparisons among various studies and locations. The hybrid test uses a combination of both ramps and steps during a single measurement. The hybrid test was chosen over a continual RPM ramp because It identifies surfaces that are not supply limited. Similar hybrid tests have been used for previous studies (Kavouras et al. 2009; Etyemezian et al. 2014; Fick et al. 2019). Following test initiation, the PI-SWERL was controlled by and all test data, including data from the DustTrak, was logged in an imbedded computer. Test operation data was monitored real-time and the test discontinued to protect the optical bench of the DustTrak if the PM_10_ concentration exceeded 400 mg m^− 3^.

**Table 1:** Summary of the hybrid PI-SWERL test used to assess surface dust emissivities and threshold friction velocities

Cumulative Test Time (s)	Interval Time (s)	Operation
0–540	540	Aspirate PI-SWERL bell and record PM_10_ concentration
0–60	60	No ring movement
61–105	45	Accelerate ring to 2000 RPM
106–165	60	Maintain ring at 2000 RPM
166–210	45	Accelerate ring to 3000 RPM
211–270	60	Maintain ring at 3000 RPM
271–315	45	Accelerate ring to 4000 RPM
316–375	60	Maintain ring at 4000 RPM
376–420	45	Accelerate ring to 5000 RPM
421–480	60	Maintain ring at 5000 RPM
481–540	60	Decelerate ring to 0 RPM

Seven sites in the Lordsburg Playa basin (Fig. 1) were tested with the PI-SWERL and at least four replicates were conducted on fresh surfaces at each of the sites. Three of the sites were located on delta deposits on the fringe of the playa associated with Animas Creek south of Interstate Highway 10 and represented land used for cattle grazing designated as sites AD1, AD2, and AD3. Two of the sites had thick, hard, clay-rich lacustrine sediments that dried into indurate crusts of distinct polygons designated sites RFP and PC. Finally, two of the sites were shoreline (beach) areas at the eastern and western margins of the lakebed designated as sites EB and WB, respectively. At each replicated test location, GPS coordinates were recorded, approximately 100 g of the 0–5 cm surface sediment was sampled, and the surface threshold friction velocity (u*_t_) was estimated using the airgun and penetrometer technique of Li et al. (2010). Where both undisturbed and disturbed (cattle tracks or vehicle tire tracks) surfaces were sampled for a total of 44 tests, GPS coordinates and soil samples were obtained. Estimates of u*_t_ using the airgun and penetrometer technique were terminated after 37 tests due to airgun malfunction. Analysis of collected airgun and penetrometer data revealed that this method may not be applicable to playa soils due possibly to the presence of soluble salts.

From PI-SWERL data, friction velocities, u* (a measure of shear-related motion in moving fluids that may result in vertical entrainment of particles) were obtained at a frequency of 1 Hz by regressing the rotating ring RPM vs Irwin sensor measured friction velocity data provided in the PI-SWERL Operator Manual v1.3 Fig. 1.2 (Dust-Quant 2011) and entering the instantaneous rotating ring RPM into the resulting regression eq. PI-SWERL estimates of threshold friction velocity u*_t_ (the friction velocity at which particles will become entrained in the fluid) were not simple; we almost always saw an initial spike in PM_10_ concentration both when the airflow started during the first few seconds of the test and again at the first few seconds of rotating ring acceleration to 2000 RPM. This spike phenomenon is possibly due to vibration within the PI-SWERL at startup, dislodging particles within the system. Under both these test phases, we saw the PM_10_ concentrations fall to near pre-test levels within 30 s after the initial spike. We visually determined the u*_t_ by inspecting the PM_10_ concentration curve to determine when the fresh PM_10_ was being entrained from the ground surface. PM_10_ emissivity expressed as mass per unit time per unit area was calculated by dividing the instrument-determined vertical flux rate data (μg s^− 1^) by the 0.026 m^2^ effective area of the rotating ring.

The 2 m mean wind velocity that would result in an exceedance of the United States EPA National Ambient Air Quality Standard (NAAQS) for PM_10_ of 150 μg m^− 3^ – representing a visibility-reducing dust cloud - was calculated by taking the measured PM_10_ concentration in the PI-SWERL that would result in a 30 m mixed column of air with PM_10_ that would equal or exceed the PM_10_ NAAQS. The height of the inside of the bell of the PI-SWERL is 20 cm. The 30 m height for mixing was arbitrarily chosen as it is quite possible in turbulence for PM_10_ to mix to this height. Thus, by multiplying the NAAQS standard by 150 times the inside depth of the bell from which dust measurements were obtained, the concentration inside the bell must be at least 22.5 mg m^− 3^ for this value to be reached or exceeded. The friction velocity calculated for this 1 s time step was subsequently entered into Prandtl’s equation using 0.4 for von Karman’s Constant and a mean value from several sources of 2 X 10^− 5^ m for the roughness length of a bare smooth surface to obtain the expected mean wind speed at 2 m. Wind speed is nearly universally reported at the standard reported height of 2 m. This would be the mean reported wind speed at which the PM_10_ NAAQS would be exceeded over similar surfaces. The same calculation based on Prandtl’s equation was used to estimate the 2 m threshold wind speed for PM_10_ entrainment. It should be noted that these wind speeds are based on a smoother bare surface as tested by the PI-SWERL and due to vegetation surrounding the bare surfaces that were tested, the actual threshold wind speeds would probably be greater than the values fit.

Weather data were collected from two NMDOT weather stations located along Interstate Highway 10 on the lakebed (Fig. 1). The stations were established on July 22, 2015 and record 15-min wind speed means and maximum gust. These data, an average of 35,064 observations per year, were compared to the estimated threshold wind speeds for PM_10_ entrainment, exceedance of the USEPA NAAQS, and the wind speed at which the maximum dust entrainment was observed during the PI-SWERL to estimate the percentage of observations exceeding these values. The last date of data considered was July 1, 2020.

The particle size distribution (PSD) of the surface soil at each site was measured by laser diffraction spectroscopy, generally following the procedures of Sperazza et al. (2004). Soil samples were passed through a 2 mm sieve to remove any gravel or plant debris and 0.2 to 0.6 g of the sieved sample was dispersed in 11.5 ml of a sodium hexametaphosphate solution. The 15 ml tubes containing the dispersed sample were shaken for 8 h before introduction to the Malvern Mastersizer 2000 (Malvern Instruments, Worcestershire, UK) that had been calibrated using 0.2 g of ISO 12103-1 A4 coarse test dust. For each dispersed sample, three individual PSD determinations were made and the means of each PSD class was calculated. From these means, we determined the percentage of sand (53 < d < 2000 μm), silt (2 < d < 53 μm), clay (d < 2 μm), and PM_10_ (d < 10 μm) in each of the soil samples.

Statistical analysis of the data was done using Proc GLM in SAS v9.4. Means were separated using Ryan’s Q. Regression relationships between the response parameters and surface factors including soil texture and % PM_10_ was performed in Microsoft Excel graphs by fitting trendlines, linear equations, and R^2^ values for the relationships.

## Results and discussion

**Fig. 4:**
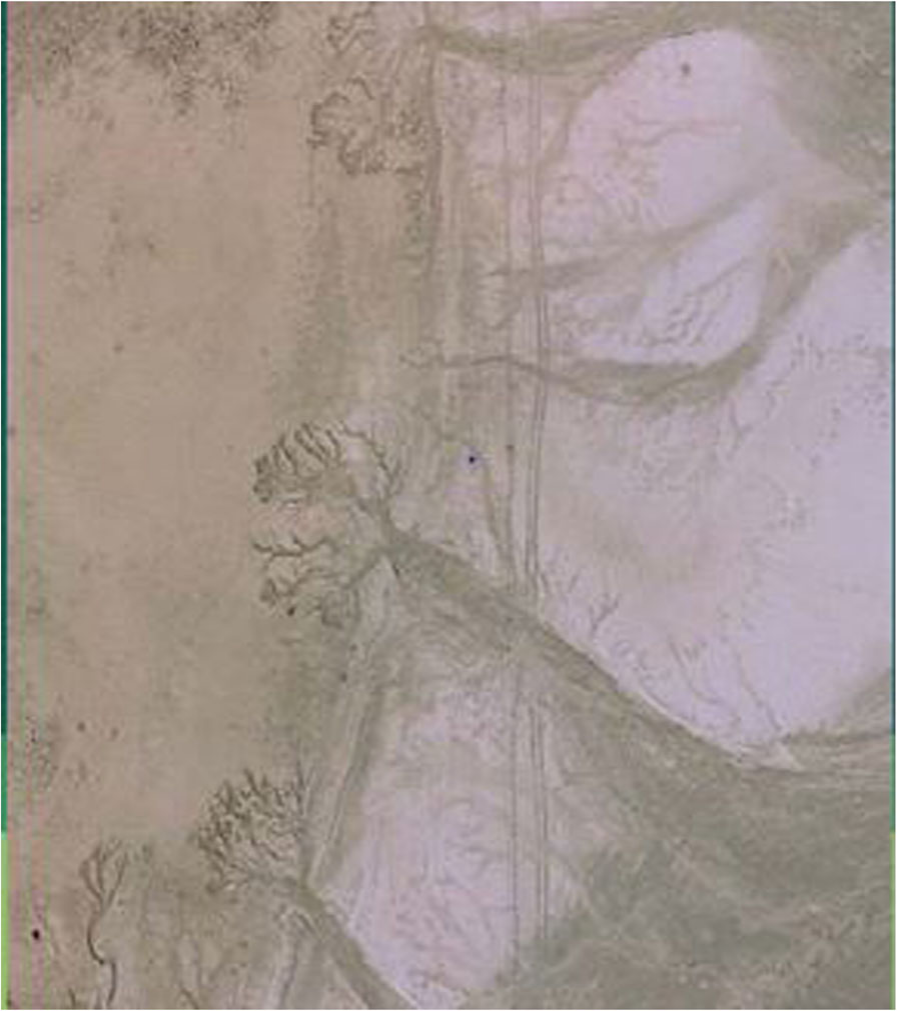
Aerial photograph of area on the west of Lordsburg Playa showing erosional head cutting in the left portions of the picture and subsequent deposition of eroded sediment on the playa surface in the right-hand portions of the image

Mean GPS locations, as well as means and standard deviations of surface sand, silt, clay, and PM_10_ percentages of each site are presented in Table 2. Although some soil PSD variation is evident among the sites, the soil at the west beach site stands out as having much sandier material and much lower content of PM_10_ than any of the others. The western edge of Lordsburg Playa is close to the Peloncillo Mountains and several small drainages empty as alluvial fans directly onto the playa surface from relatively steep terrain. In addition to the nearby steep terrain and resulting coarse sediment transport on the west side of the playa, close examination of aerial photography, satellite images, and discussions with local land managers (Trent Botkin, NMDOT, personal communication) has revealed that the dams of several small, decades-old impoundments related to mining and cattle grazing on the mountain slopes have been breached. These impoundments filled with sediment over time and when their dams breached during seasonal rains, massive coarse sediment flows were often formed which in this area have flowed far onto the lakebed surface (Fig. 4). Prior systematic analyses of remote sensing imagery have suggested that these zones of contact between coarse sediments and fine playa materials play an enhanced role in initiating dust storms in the Chihuahuan Desert due to the ability of the coarse sandy materials to saltate (hop in the wind) with resultant sandblasting of the finer, wind-entrainable lacustrine sediments (Rivera-Rivera et al. 2010).

**Table 2:** Mean and standard deviation of longitude, latitude, percent sand, silt, clay and PM_10_ for the surface sediment samples at the test sites

Site (Surface Class)^a^		Longitude	Latitude	Sand	Silt	Clay	PM_10_
		Degrees W	Degrees N	%	%	%	%
AD1 (D)	Mean St. Dev.	108.854 1.91 E-5	32.255 8.64 E-5	17.79 1.01	69.2 1.10	12.29 0.50	41.30 1.36
AD2 (D)	Mean St. Dev.	108.868 3.86 E-5	32.254 3.47 E-5	28.64 4.94	57.42 4.16	13.94 3.16	42.25 7.40
AD3 (D)	Mean St. Dev.	108.878 8.03 E-5	32.267 5.4 E-5	18.01 1.08	70.10 1.29	11.90 1.32	45.24 3.41
AD2 (L)	Mean St. Dev.	108.868 1.04 E-4	32.254 3.98 E-5	30.66 5.37	57.16 7.05	12.18 1.67	37.57 2.62
RFP (L)	Mean St. Dev.	108.943 5.89 E-4	32.242 6.68 E-4	29.02 12.27	62.27 12.00	8.70 0.51	33.72 2.93
PC (L)	Mean St. Dev.	108.908 3.18 E-4	32.322 2.47 E-4	17.52 3.88	54.62 1.95	27.85 2.81	57.73 4.55
RFP (B)	Mean St. Dev.	108.944 --	32.242 --	56.72 --	36.17 --	7.11 --	25.22 --
EB (B)	Mean St. Dev.	108.832 1.65 E-4	32.333 7.11 E-5	41.33 7.13	45.35 6.13	13.32 1.70	42.55 7.06
WB (B)	Mean St. Dev	108.933 1.93 E-5	32.315 4.41 E-5	84.59 13.00	12.23 10.76	3.17 2.24	10.80 8.83

^a^surface classes are D = Delta, L = Lake, and B = Beach

Test site surface type, disturbance class, as well as friction velocities at threshold (u*_t_), NAAQS exceedance (u*_exc_), and maximum PM_10_ flux (u*_max_), as well as the maximum vertical flux rate (MaxQ) and total PM_10_ flux (TotQ) are presented in Table 3. In general, the sites we tested in the Animas Creek delta (D) and in the beach areas along the shorelines (B) were much more emissive based on MaxQ and TotQ than lakebed sites at RFP and PC that were ephemerally flooded (L). Specifically, the undisturbed delta sites had MaxQ values between 3 and 7 times and TotQ values between 3 and 6 times the mean value for the ephemerally flooded sites. The disturbed sites had MaxQ values between 26 and 59 times greater and TotQ values between 26 and 67 times the mean value for the ephemerally flooded sites. Sites in the delta to the south, AD1 and AD2, had apparently been cultivated at some time in the past, but all were being used for cattle grazing at the time of the field investigation. At one of the three sites, AD2, furrows had been cut between the natural soil surface to hold water and, hopefully, increase the survival of grass seedlings. The surfaces of these furrow bottoms were very similar to the hard crust polygons found in ephemerally flooded areas and their emission rates were also very similar. For this reason, we considered them to be ephemerally flooded and segregated them from the other tests at this site. In a similar manner, one of the tests at the Road Forks Playa (RFP), an isolated ephemerally flooded area where Interstate Highway 10 enters Lordsburg Playa from the west, was performed on a mantle of aeolian sand that resembled a beach deposit and so we classified that as a beach type surface. With these exceptions, the replicate spots in each site were very uniform in surface characteristics and all PI-SWERL test derived data were used to calculate the means presented in Table 3.

**Table 3:** Surface class (Delta, Lake, or Beach), disturbance class (Undisturbed or Disturbed), and mean and standard deviation of threshold friction velocity (u*_t_), friction velocity at which the NAAQS standard would be exceeded in a 30 m tall column of air (u*_exc_), friction velocity at which the maximum rate of PM_10_ vertical flux is observed (u*_maxQ_), the maximum rate of PM10 vertical flux observed (Max Q), and the total PM_10_ vertical flux for the nine minute PI-SWERL test (Tot Q) of each sample site

Site	Surf. Class	Disturb. Class		u*_t_ (m s^−1^)	u*_exc_ (m s^−1^)	u*_maxQ_ (m s^−1^)	Max Q (μg m^−2^ s^− 1^)	Tot Q (μg m^−2^)
AD1	D	U	Mean St. Dev.	0.31 0.02	0.80 0.02	0.81 0.00	1325.48 919.87	84,217 69,106
AD1	D	D	Mean St. Dev.	0.31 0.02	0.62 0.07	0.81 0.01	11,030.94 5498.17	845,554 5,168,485
AD2	D	U	Mean St. Dev.	0.26 0.05	0.74 0.12	0.80 0.02	3098.75 2021.42	193,221 131,854
AD3	D	U	Mean St. Dev.	0.24 0.05	0.60 0.15	0.80 0.02	1248.26 8697.52	999,201 832,504
AD3	D	D	Mean St. Dev.	0.31 0.02	0.50 0.03	0.68 0.05	24,977.63 1778.07	2,226,249 682,823
AD2	L	U	Mean St. Dev.	0.31 0.01	-- --	0.80 0.00	223.60 36.49	16,733 2287
RFP	L	U	Mean St. Dev.	0.36 0.06	-- --	0.81 0.00	476.06 406.79	45,648 77,958
PC	L	U	Mean St. Dev.	0.56 0.10	0.81 --	0.80 0.02	561.84 717.75	36,857 49,034
RFP	B	U	Mean St. Dev.	0.39 --	0.77 --	0.81 --	2450.36 --	288,993 --
EB	B	U	Mean St. Dev.	0.30 0.10	0.72 0.08	0.81 0.00	7561.78 12,282.01	788,012 1,266,773
WB	B	U	Mean St. Dev.	0.28 0.04	0.50 0.05	0.80 0.02	17,182.49 6844.97	2,727,300 1,084,141

Weather data indicated that March, April, and May have greater 15-min mean wind speeds than the other months. This is very typical of the spring winds in southwestern North America and represents the season with the greatest number of synoptic dust storms covering large areas. When gusts are considered, however, frequencies of exceedance for the threshold wind speeds increase for all months, especially during June, July, and August, months in which afternoon convective storms may form. In general, December, January, February, and September are the months with the calmest winds.

Surface physical characteristics at the test sites such as texture, structure, and the presence or absence of an undisturbed crust had a significant impact on u*_t_ (*p* < 0.0001), but not u*_exc_ (*p* = 0.1658) or u*_max_ (*p* = 0.7975). PM_10_ emissions on the other hand were greatly affected by surface physical characteristics with the mean MaxQ of the beach deposits being more than 2 times that of undisturbed delta soil surfaces whose mean MaxQ was in turn over an order of magnitude greater than the ephemerally flooded lacustrine deposits (*p* = 0.0018). Similarly, the TotQ during the test was highly influenced by surface physical characteristics with the mean TotQ of beach deposits being over three times greater than the mean for undisturbed delta soil surfaces which had mean TotQ of more than an order of magnitude greater than the mean for ephemerally flooded lacustrine deposits (*p* = 0.0003).

Although disturbance only influenced one of the u*, specifically u*_exc_, values significantly, it had a very significant effect on the maximum vertical flux of PM_10_ (MaxQ) during the testing with disturbed sites having mean emission rates of nearly an order of magnitude greater than the undisturbed sites (*p* = 0.0131). Similarly, the total PM_10_ emitted by the disturbed sites was more than an order of magnitude greater for the disturbed sites than the undisturbed (*p* = 0.0266). These trends of increased PM_10_ emissions with soil crust disturbance are consistent with experimental findings at other wind erodible playas (Cahill et al. 1996; Baddock et al. 2011b) and desert surfaces (Van Pelt et al. 2017; Klose et al. 2019) in the North American drylands, specifically including the effects of cattle activity (Baddock et al. 2011b) and off-highway vehicles (Goossens and Buck 2009) in increasing dust emission through breakage of surface crusts. The variance among disturbed sites for both these measures was much greater than for undisturbed sites and thus we can state that undisturbed sites were more predictable in their emission rates and total emissions than disturbed sites. This indicates the necessity of limiting disturbance of natural dryland soil crusts in land management plans for mitigating the dust hazard, especially in areas devoid of vegetation where aerodynamic roughness lengths are very small.

In general, the three sites on the Animas Creek delta, AD1, AD2, and AD3, had soils that were fine textured with shallow surface crusting. At site AD1, the movement of cattle along a fence line had disturbed the surface very close to undisturbed PI-SWERL replicate test spots and we tested them as a subset of the site tests. Similarly, at site AD3, side by side undisturbed and tire-track-disturbed surfaces allowed us to assess the effects of crustal disturbance on PM_10_ emissions and related critical friction velocity parameters. We found no differences in u*_t_ related to disturbance of the soil crust at either AD1 or AD3 (*p* = 0.9797) but the friction velocities at which the NAAQS standard of 150 μg m^− 3^ would be exceeded in a 30 m atmospheric column, u*_exc_, was significantly greater for undisturbed soil crusts than for disturbed (*p* = 0.0305). The mean values of u*_exc_ for the disturbed spots tested at AD1 and both undisturbed and disturbed spots at AD3, when adjusted to 2 m wind speed equivalents, were among very few with less than gale force (< 17.5 m s^− 1^) velocities although frequencies of exceedance during the five years of wind speed data collection were only about 0.2% and 1.4%, respectively for maximum wind gusts and much less than that for 15-min means. The undisturbed surface crust at AD3 was very fragile and during a PI-SWERL test of these spots, it was not uncommon to see a very rapid rise in PM_10_ vertical flux rates. Such rapid increases would be consistent with the visual observation at the end of a test where the shear force had displaced a polygon of crust and revealed easily entrainable sediments below, which has been noted as a key process threshold for increasing dust emission at other playas (Cahill et al. 1996). Disturbance also did not significantly influence the friction velocities at which the maximum vertical flux was observed, u*_max_ (*p* = 0.6491) although the variances of all friction velocity values were much smaller for the undisturbed sites demonstrating the stabilizing influence of soil crusting. We estimate from 15-min mean wind speeds that the threshold wind speed at which PM_10_ entrainment would be initiated would be exceeded approximately 5% of the time for sites at AD1, the Lakebed surface at AD2, and the disturbed surface at AD3. Other delta surface sites had higher frequencies of exceedance including approximately 10% of the time for the undisturbed delta surface at AD2 and 13% of the time for the undisturbed surface at AD3. When maximum gusts are considered, the frequencies of exceedance for threshold wind speed for PM_10_ entrainment approximately double. Only the AD3 disturbed site had 2 m U_maxQ_ values low enough that gusts have exceeded this wind speed more than once or twice in the last five years but this is a notable artefact of early test terminations to protect the optical bench of the nephelometer.

**Fig. 5:**
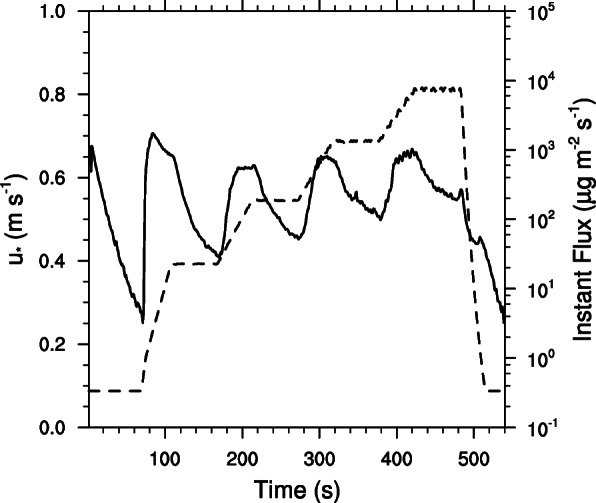
PI-SWERL test response curve showing PM_10_ supply limitations. The dashed line represents the friction velocity (u*) and the solid line is the instantaneous vertical flux of PM_10_. This test was from an area that is ephemerally flooded (L)

Naturally crusted (undisturbed) spots at site AD2 had lower emission rates and totals than the undisturbed spots at AD1 and AD3. However, the ephemerally flooded furrows had emission rates and totals an order of magnitude smaller than the naturally crusted surfaces. These furrows had highly indurate polygonal crusts very similar to the ephemeral lakebeds. Although it was less evident with the disturbed site tests, there is a limitation of PM_10_ supply in the soils of the Animas Creek delta. This is shown in Fig. 5 by a drop of emission rate following each local maximum as the RPM and resulting shear force on the surface caused by the PI-SWERL rotating ring was increased and then held steady.

**Fig. 6:**
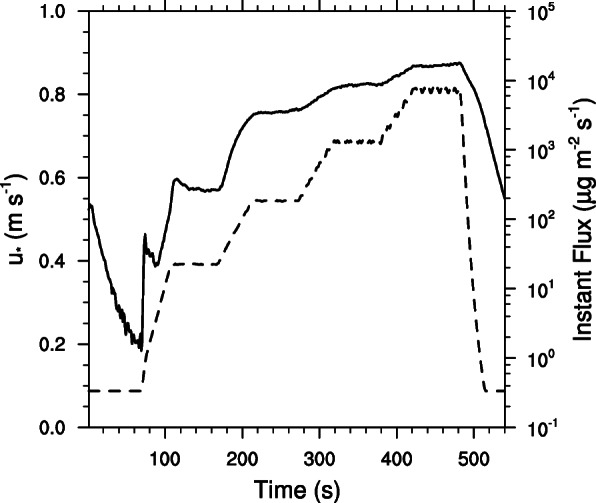
PI-SWERL test response curve showing no PM_10_ supply limitations once threshold friction velocity (u*_t_) has been exceeded. The dashed line represents the friction velocity (u*) and the solid line is the instantaneous vertical flux of PM_10_. This test was from the western beach area (WPL)

Playa beach deposits at the eastern (EB) and western (WB) ends of the pipeline service road that bisects the large playa surface just north of the highway were tested and found to be highly emissive, especially the WB. The u*_t_ means of these sites were not significantly different (*p* = 0.6630) and were about the same as those found for undisturbed surfaces in the Animas Creek delta but exhibited greater variance. The 2 m 15-min mean wind speeds exceeded the estimated threshold for PM_10_ entrainment at EB and WB approximately 6% and 8% of the time periods respectively and when gusts were considered, the threshold was exceeded approximately 15% and 18% of the time respectively. The mean u*_exc_ for WB was significantly lower than that for EB (*p* = 0.0112). At EB, the 2 m 15-min mean wind speed that would exceed the threshold for NAAQS levels would occur less than 0.01% of the time and for WB approximately 0.16% of the time and for gusts the threshold would be exceeded 0.03% and 1.37% of the time respectively. In spite of the significantly lower u*_exc_ for WB, the mean u*max for WB, although much more variable, was not significantly lower than the mean for EB (*p* = 0.3847). Although the mean maximum PM_10_ flux rate for WB was more than twice that for EB, high variance at both sites precluded any significance in the difference (*p* = 0.2202). More side of the playa effect was found for the mean total flux of PM_10_ (TotQ) in which the mean total for WB is nearly four times that for EB (*p* = 0.0589). The primary test response difference between EB and WB was that the tests conducted at WB did not exhibit any apparent supply limitation (Fig. 6) and maintained their emission rates until the RPM and resultant shear stress of the PI-SWERL rotating ring increased to the next level and was held. At EB, supply limitation was apparent at lower values of friction velocity at the early time steps of the test and only overcame supply limitation at higher values of friction velocity. Like many of the test surfaces on the Animas Creek delta, the spots tested at WB also had a mean value of u*_exc_ that, when converted to 2 m wind speed equivalents, was also less than gale force. Events with 2 m wind speeds in excess of gale force for extended periods of time are rare at inland mountainous locations. Thus, most of the dust entrained will, with the exception of gusts or short periods in which strong pressure gradients are being equilibrated, be entrained with less than gale force velocities.

The two sites tested that represent the ephemerally flooded lakebed included the small Road Forks Playa (RFP) just south of the highway near the western edge of the Lordsburg Playa and a site north of the pipeline access road very near the center of the northern half of the large Kathrine Playa (PC). At the RFP test site, four ephemerally flooded surfaces were tested and one that had a mantle of aeolian sand that we classed as a beach type surface. At the PC test site, nine tests were conducted. Values of mean observed u*_t_ were more than 50% greater than for the beach and delta deposits. The tests conducted on the ephemerally flooded surface at RFP did not result in a value of u*_exc_ that would have exceeded the NAAQS standard for PM_10_. From weather station data, the 2 m 15-min mean wind speed would have exceeded the threshold for PM_10_ entrainment at the RFP and PC test sites 2.3% and 0.02% of the time respectively and considering gusts, the frequencies would increase to 8.3% and 0.6% of the time respectively.

At the PC site near the center of the playa, only one of the nine replicate test spots resulted in a NAAQS standard exceedance for PM_10_ in a 30 m column of atmosphere and that value was near the maximum of friction velocities recorded during all phases of testing at all sites. The 2 m equivalent wind speed would have exceeded 23 m s^− 1^, and this wind speed was only exceeded by maximum gusts twice in the five years of record. The mean value of u*_max_ for these surfaces was slightly lower than that noted for the other two surface types and had greater variance. The mean maximum vertical flux of PM_10_ from ephemerally flooded surfaces was less than 500 μg m^− 2^ s^− 1^ and the mean total PM_10_ flux during the nine-minute test was less than 37 mg m^− 2^.

Although the ephemerally flooded lacustrine deposits occupy the predominant surface area in the complex Lordsburg Playa landscape, at least during our March 2018 testing, they exhibited lower PM_10_ emissivities than would be necessary to obscure visibility along Interstate Highway 10. This is due to the limited abrader material on the surface. Sand from beach areas or massive coarse sediment flows saltating downslope and downwind across large areas of the indurate clay-rich crust or disturbance by crust crushing activities such as off-road vehicle traffic or cattle grazing could potentially change this situation. One of the limitations of the PI-SWERL is the inability to introduce abrader sand and this limitation may have limited PM_10_ emission observations. The trampling of crusts by the movement of cattle was demonstrated to increase dust emissions during controlled wind tunnel tests on another playa in southern New Mexico (Baddock et al. 2011b) and it would likely do so at Lordsburg Playa. Lordsburg Playa has been closed by the United States Bureau of Land Management to off-highway vehicle use since 1998 ‘to reduce impacts to the soil on the Lordsburg Playa. Once the soil surface is disturbed, it is highly susceptible to wind erosion’ (United States Department of the Interior 1998). Observations of the authors and New Mexico state employees suggest that unauthorized crust crushing off-road recreational vehicle use still takes place on the playa surface. Rigorous monitoring and enforcement of this prohibition should be increased, since even a small area of disturbed playa in the wrong place and an unfortunate gust of wind could initiate a dust plume crossing the highway and dangerously reducing visibility.

Mitigation of the visibility hazard on Interstate Highway 10 caused by the current active dust emission sources will require considerable expense for revegetation and disruption to agricultural activities in the immediate region. The dust sources occur on a spatial matrix of state, federal, and private land, with each type of ownership having unique legal and management considerations. Grazing leases on public land may be retired by the managing agencies, but privately held land will probably still be used for grazing with little restriction. Much of the publicly-owned lands are leased to local landowners to supplement the grazing land they use for their livestock. The loss of these additional lands will result in a financial hardship to these producers that cannot be easily mitigated. In a similar way, prohibition of off-road vehicle use may be mandated on public land, but enforcement of the mandate will not be without problems including the landowner access to private land inholdings (otherwise surrounded by state and federal land). Although these changes to land use will create negative economic impacts to the local agricultural community, the costs will pale in comparison with the costs of loss of property, life, health and safety, and disruption of the transportation infrastructure that is currently inherent with the visibility related crashes on Interstate Highway 10 at Lordsburg Playa. Costs exceeding US $ 1 million may be estimated per loss of human life related to medical care, emergency services, productivity over a lifetime, insurance, workplace costs, and legal costs (Blincoe et al. 2015). A simple calculation of economic costs alone in terms of financial impacts cannot take into account the effects on others, including family and friends who depend on that person for physical support, emotional support, and quality of life.

## Conclusions

Lordsburg Playa in New Mexico, USA is representative of playa (dry lakebed) environments in many desert regions where windblown dust emissions create traffic safety and crash hazards, and a priority site for environmental remediation to improve highway safety and reduce detour-related delays and losses. We tested the surface emissivity characteristics at sites representing different landscape positions, sedimentology, management histories, and surface crust disturbance at Lordsburg Playa using a PI-SWERL. A wide range of values for threshold friction velocity, friction velocity at which the National Ambient Air Quality Standard would be exceeded in a 30 m column of air (representing formation of a dust cloud), PM_10_ (fine dust) vertical flux rates, and total PM_10_ vertical flux during the nine minute test were indicative of the complexity of the surfaces encountered in the immediate vicinity of Lordsburg Playa. We also found that the critical friction velocities and emissivities were not strongly dependent on the surface sediment texture but were highly dependent on surface crust strength, thickness, and disturbance.

From our data, we concluded that the actual lake bed surface is not strongly dust-emissive when intact even when dry, but the shoreline margins or beach areas and areas of the Animas Creek delta are highly dust-emissive and should be the focus areas of management to mitigate wind erodibility including limiting disturbance and augmenting native sediment trapping vegetation when possible. The western shoreline of the playa was the most emissive of the sites tested and was one of the few areas without dust supply limitation even though the surface sediments contain by far the lowest percentage of PM_10_; this is likely due in part to legacy sediment control structures (dams and berms) on the slopes above the playa which are failing and releasing pulses of debris onto the western playa surface. For these reasons, this area should be a priority for dust emission control measures followed by areas on the Animas Creek delta that develop more fragile crusts. In order to improve highway safety and reduce the risk of continued visibility-related crashes and shutdowns, the playa should be carefully monitored for and protected against activities that disturb the crust and increase dust emissions. Plans are currently being considered for remediation of the dust sources on and near the Lordsburg Playa (Botkin and Hutchinson 2020). It is hoped that by identifying the most emissive sources, priority areas can be identified that will rapidly result in mitigation of the dust visibility hazard along this section of Interstate Highway 10.

## Data Availability

The authors will provide the data upon request.

## Abbreviations

AD1: PI-SWERL test Site 1 on the Animas Creek Delta
AD2: PI-SWERL test Site 3 on the Animas Creek Delta
AD3: PI-SWERL test Site 3 on the Animas Creek Delta
ADOT: Arizona Department of Transportation
B: Beach deposit, usually sandy material above the playa flood pool
BLM: U.S. Department of the Interior Bureau of Land Management
D: Delta deposits from Animas Creek, mostly south of Interstate Highway 10
EB: PI-SWERL test site at the east end of Pipeline Road (a beach deposit)
GPS: Global Positioning System
L: Lacustrine deposits in ephemerally flooded areas of the playa, usually clay-rich and highly indurated
MaxQ: The greatest 1 s vertical flux of PM_10_ during a PI-SWERL test, an index of surface emissivity
NAAQS: U.S. Environmental Protection Agency National Ambient Air Quality Standards
NMDOT: New Mexico Department of Transportation
NRCS: U.S. Department of Agriculture Natural Resources Conservation Service
PC: PI-SWERL test site near the center of the ephemerally flooded playa
PI-SWERL: Portable In-Situ Wind ERosion Laboratory
PM_10_: Particles with diameters less than ten micrometers
PSD: Particle Size Distribution
RFP: PI-SWERL test site at the Road Forks Playa, an ephemerally flooded surface south of Interstate Highway 10
SAS: Statistical Analysis Software published by the SAS Institute
TotQ: The total PM10 vertical flux produced during the 540 s PI-SWERL test
u*: Friction velocity
u*_exc_: Friction velocity at which sufficient PM_10_ vertical flux was measured to exceed NAAQS
u*_max_: Friction velocity at which the greatest vertical flux of PM_10_ was measured
u*_t_: Threshold friction velocity, the friction velocity at which vertical flux of PM10 was initiated
WB: PI-SWERL test site at the west end of Pipeline Road (a beach deposit)
